# Efficacy of sorafenib in BRAF-mutated non-small-cell lung cancer (NSCLC) and no response in synchronous BRAF wild type-hepatocellular carcinoma: a case report

**DOI:** 10.1186/s12885-016-2463-2

**Published:** 2016-07-07

**Authors:** Andrea Casadei Gardini, Elisa Chiadini, Luca Faloppi, Giorgia Marisi, Angelo Delmonte, Mario Scartozzi, Cristian Loretelli, Alessandro Lucchesi, Devil Oboldi, Alessandra Dubini, Giovanni Luca Frassineti, Paola Ulivi

**Affiliations:** Department of Medical Oncology, Istituto Scientifico Romagnolo per lo Studio e Cura dei Tumori (IRST) IRCCS, 47014 Meldola, Italy; Biosciences Laboratory, Istituto Scientifico Romagnolo per lo Studio e Cura dei Tumori (IRST) IRCCS, Meldola, Italy; Department of Medical Oncology, AO Ospedali Riuniti, Università Politecnica delle Marche, Ancona, Italy; Department of Medical Oncology, University of Cagliari, Cagliari, Italy; Radiology Unit, Istituto Scientifico Romagnolo per lo Studio e Cura dei Tumori (IRST) IRCCS, Meldola, Italy; Pathology Unit, Morgagni-Pierantoni Hospital, Forlì, Italy

**Keywords:** Case report, BRAF, HCC, NSCLC, Sorafenib

## Abstract

**Background:**

Sorafenib is a multi-targeted kinase inhibitor with a demonstrated activity in renal cell carcinoma (RCC) and hepatocellular carcinoma (HCC), and it is currently used for the treatment of these pathologies. Ongoing clinical trials are studying its activity in other malignancies, such as non-small-cell lung cancer (NSCLC). However, no biological marker is known to define either the sensitivity or resistance to the drug.

**Case presentation:**

Here we report a case of a patient with two synchronous tumors, HCC and NSCLC, with metastases in the contralateral lung and bone. The patient was treated with gemcitabine as first line, with a resulting progressive disease after two months, and then with sorafenib at standard dosage in the second line setting. After 6 months of treatment CT scan showed a partial response in the primary lesion of the lung, complete response of the metastasis in the contralateral lung, and stability of HCC. The patient had progression in the lung, liver and bone after 13 months of therapy. A molecular characterization of NSCLC and HCC lesions was performed, revealing a *BRAF* exon 11 mutation (G469V) only in NSCLC. We hypothesize that the response observed in NSCLC lesions could be due to the presence of *BRAF* mutation, and that this alteration could be responsible in determining sorafenib sensitivity.

**Conclusions:**

Results observed in this case encourage further research on the activity of sorafenib in both HCC and NSCLC, based on the presence of *BRAF* mutation. This could lead to a selection of HCC patients to be treated with this drug, and could help identify a novel treatment strategy for *BRAF*-mutated NSCLC patients.

## Background

Sorafenib is a multi-targeted kinase inhibitor with proven activity in renal cell carcinoma (RCC) and hepatocellular carcinoma (HCC) [1, 2]. It was originally discovered as an inhibitor of Raf-1 kinase, but was found to have an expanded target profile with potent activity against other kinases including BRAF, vascular endothelial growth factor receptor (VEGFR)-1, VEGFR-2, VEGFR-3, platelet derived growth factor (PDGFR)-β, KIT, Flt-3, and RET. It has a broad-spectrum efficacy in human tumor xenograft models including NSCLC [3, 4].

NSCLC seemed an ideal disease in which to further investigate sorafenib based on the frequency of *RAS* mutations, particularly in adenocarcinomas [5–7]. Several clinical trials have evaluated sorafenib in the treatment of advanced NSCLC alone or in combination with chemotherapy or targeted agents, without reaching consistent results on efficacy [8–11]. Markers of sorafenib efficacy or resistance have yet to be identified [12–15].

## Case presentation

**Fig. 1:**
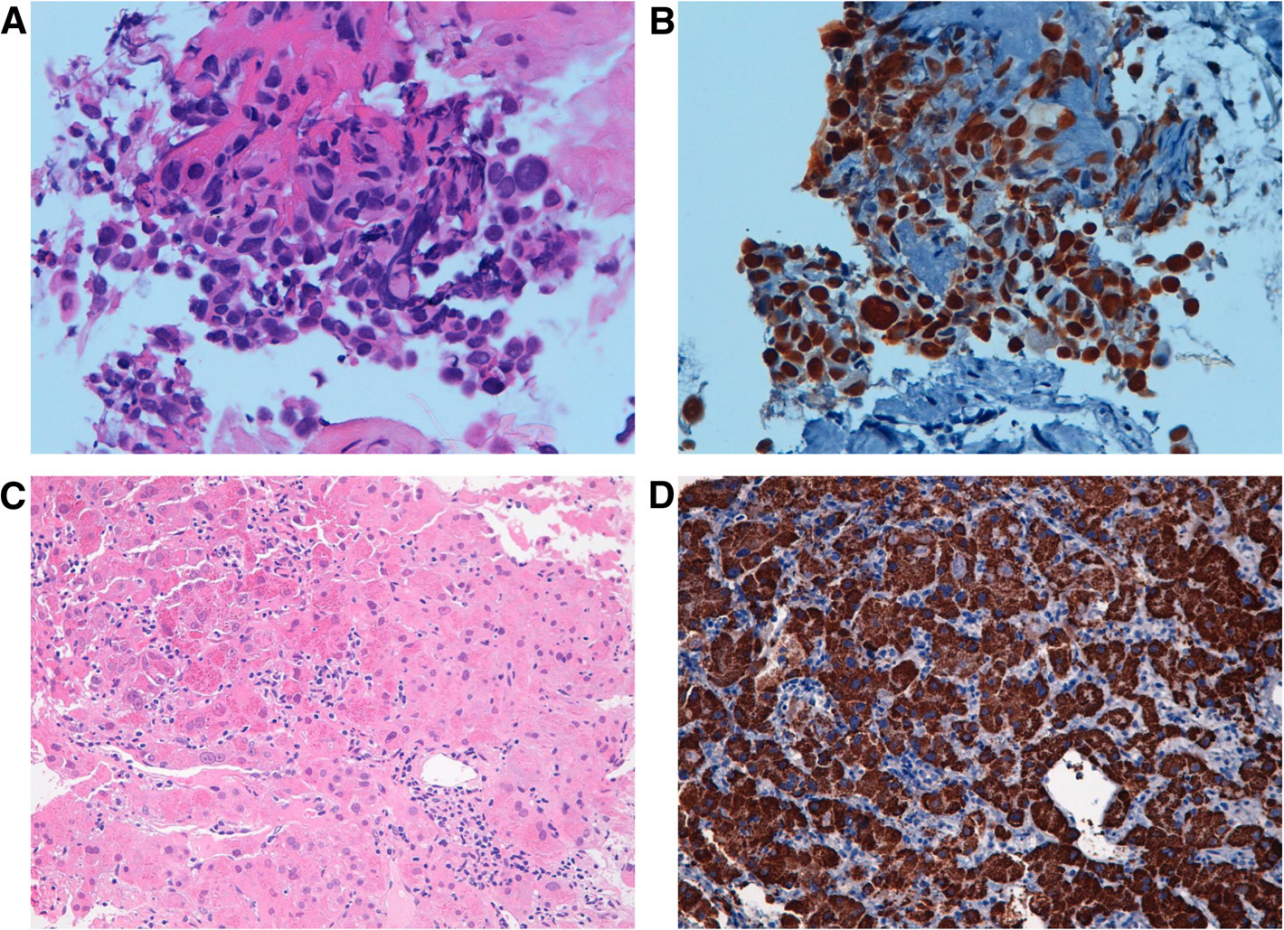
Lung: high power view of aggregate of primary lung adenocarcinoma cells **a**, with diffuse and intense nuclear staining for Thyroid transcription factor-1 (TTF-1). **b**. Liver: high power view of HCC, **c**, with a distinct granular cytoplasmic staining for HepPar-1, **d**

We present a case of a 74-year-old man smoker patient with NSCLC with bone metastases (T2NXM1) and HCC (BCLC stage C). The patient had a related liver cirrhosis metabolic syndrome, good liver function (Child Pugh A5), and reported a diabetes mellitus type II in his past medical history. In July 2014 for chest and abdominal pain he performed a CT scan with evidence of lung and liver lesions, and bone metastasis. Lung biopsy performed on primary lung lesion showed pulmonary adenocarcinoma (TTF1 positive and p40 negative) (Fig. 1a-b) and liver biopsy showed HCC (grade 2 Edmondson) (Fig. 1c-d). As the patient was not in good clinical conditions due to grade 2 asthenia, we decided to start with gemcitabine in monochemotherapy in August 2014. After 2 months of chemotherapy a further CT scan showed a disease progression in both the lung and the liver. We decided to initiate treatment with sorafenib with standard schedule (400 mg bid continuously).

**Fig. 2:**
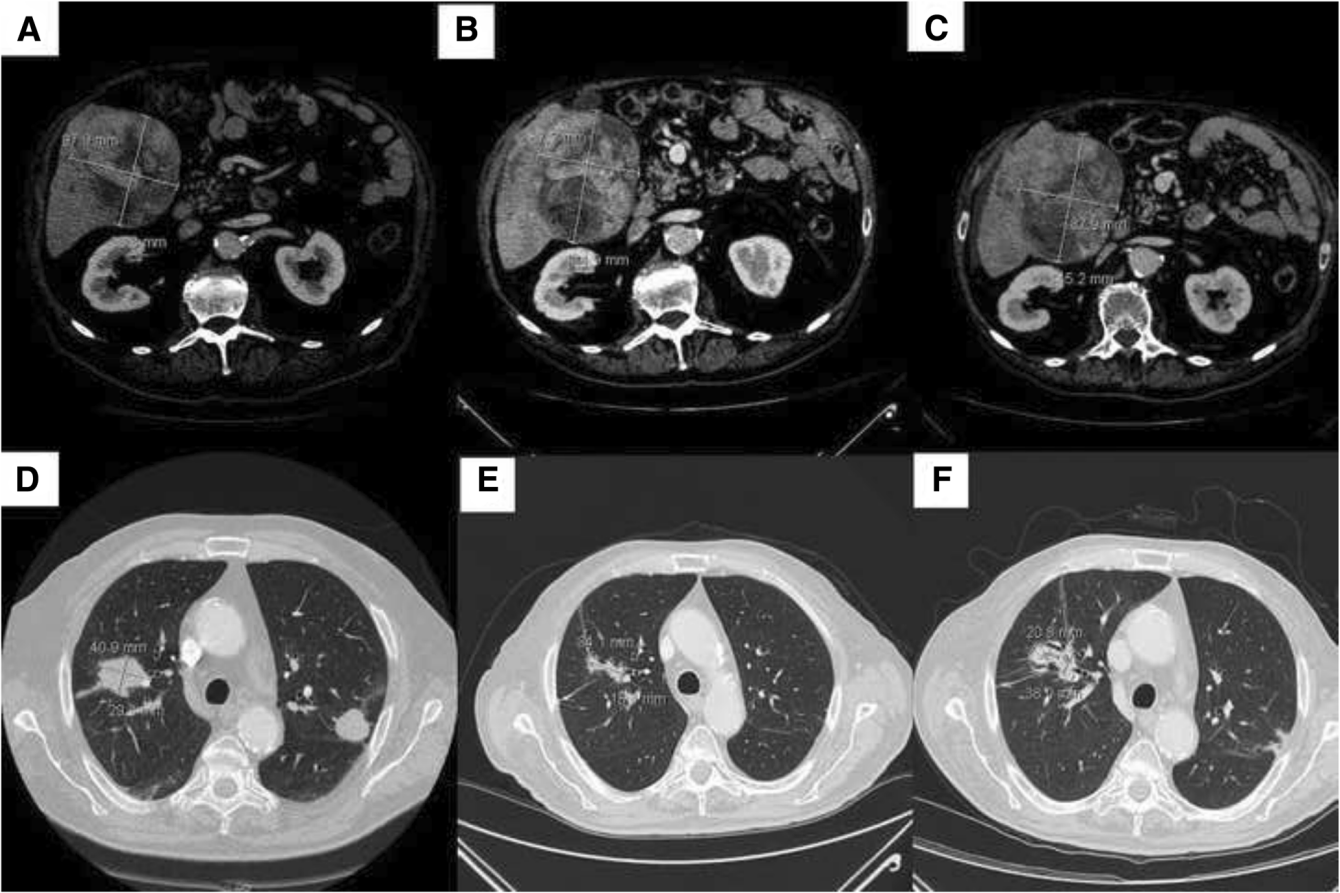
CT scan confirmed the presence of liver, **a** and lung lesions, **d**. CT re-evaluation after 6 months showed stability of HCC, **b**, a partial response in the right lobe of the lung and a complete response in the left lobe of the lung, **e**. Disease progression in the lung and liver, **c**-**f**

CT scan before therapy showed that the primary liver lesion measured 97 mm × 98.3 mm (Fig. 2a). The primary lung lesion measured 40.9 mm × 29.3 mm (Fig. 2d) and the metastasis in the contralateral lung measured 27 mm × 25 mm (Fig. 2d). After 20 days we decided to reduce the dose of sorafenib to 400 mg per day for adverse events (hypertension grade 2 and mucositis grade 3). This dose was maintained until progression, without adverse events. CT scan after 2 months showed partial response in both lung lesions and stable disease in the liver and bone lesions. CT scan after 6 months of therapy showed partial response of the primary lung lesion and complete response of the lung metastasis (Fig. 2e). HCC was stable (Fig. 2b). After 13 months of therapy CT scan showed a disease progression in both the lung and the liver (Fig. 2c-f). Due to poor performance status of the patient we decided to treat patient with only best supportive care.

The pulmonary lesion underwent routine diagnostic molecular characterization for *EGFR*, *KRAS*, NRAS, *PIK3CA*, *BRAF*, *ERBB2*, *ALK*, *DDR2*, *MAP2K1*, *RET* mutations using Myriapod Lung Status (MassARRAY Sequenom). Results revealed an exon 11 point mutation on *BRAF* gene (G469V).

The same analysis was performed on the liver lesion, with no mutations in the different genes. Genomic DNA extraction from both lesions was performed starting from tumor sections composed of about 70 % of tumor cells.

Taking into consideration our previous results obtained in HCC patients, in which we have demonstrated that specific polymorphisms of *eNOS*, *VEGFA*, *VEGFC* and *HIF*-*1alpha* seem to correlate with response to sorafenib [16–18], we performed the analysis of such polymorphisms on our patient. Results showed an homozygous status for *eNOS* VNTR (4bb) and *HIF*-*1alpha* rs12434438 GG. Both of these polymorphisms were associated with a worse prognosis in our previous studies [16–18].

The molecular determinations performed on the liver lesion (not part of routine molecular diagnostics) and the polymorphism analyses, both part of an ongoing research protocol on liver cancer approved by our Local Ethics Committee, were carried out after obtaining written consent from the patient.

## Conclusions

In this case report we showed that *BRAF*-mutated tumors could be responsive to sorafenib. Results of recent studies have shown an activity of anti-BRAF agents, such as vemurafenib and dabrafenib, in *BRAF* V600E-mutated NSCLC patients [19, 20]. In a retrospective study performed on *BRAF* mutated NSCLC patients receiving anti-BRAF treatment outside clinical studies, one patient was treated with sorafenib showing a partial response [21]. Very few data are present in the literature on the role of *BRAF* non-V600E mutations in determining the type of response to anti-BRAF agents in NSCLC. A case report of a patient with *BRAF* G469L demonstrated absence of response to vemurafenib [22]. Conversely, another case report of a patient with lung adenocarcinoma harboring *BRAF* G469R mutation, showed a strong and rapid response to sorafenib [5] for up to 6 months. Moreover, a recent case report demonstrated a strong and durable response to sorafenib in a patient with lung adenocarcinoma carrying an *ARAF* (p. S214C) mutation [23], suggesting the potential of this drug in treating patients with alterations in this pathway. No clinical studies have evaluated the role of sorafenib in BRAF mutated NSCLC patients, and clinical trials on sorafenib in unselected patients with advanced NSCLC have demonstrated modest activity, with no survival advantage [7, 10]. A phase II study evaluated the activity of sorafenib in an unselected NSCLC case series. In this study, performed on 34 patients, 2 partial responses and 20 stable diseases were observed, without correlation with neither *KRAS* nor *EGFR* statuses. However, *BRAF* status was not determined [24].

We report a case showing efficacy of sorafenib in one NSCLC patient carrying an exon 11 G469V *BRAF* mutation. The patient, treated with sorafenib for synchronous HCC, showed a good response in lung lesions, carrying the *BRAF* mutation, whereas no response was observed in the hepatic lesion, which was *BRAF* wt. Conflicting results are found in the literature on the frequency of *BRAF* mutation in HCC, as about 20 % of HCC mutated in one Italian study [25], whereas no or a very low mutation rate was observed in other studies [26, 27]. However, no results are reported on the correlation between *BRAF* mutation and sorafenib response in this pathology. Results of this case report seem to suggest that sorafenib activity could be more evident in lesions carrying a *BRAF* mutation (lung lesions in this case), with respect to the *BRAF* wt lesion (hepatic lesion). Although no correlation has been observed between sorafenib and *KRAS* mutation, the association with *BRAF* mutation remains to be established. As biochemical assays, performed to demonstrate the activity of the drug on the different components of RAF/MEK/ERK pathway, showed that the higher activity of sorafenib is evident against CRAF and BRAF (both wt and mutant) proteins [28], we hypothesized that sorafenib could be effective in *BRAF* mutated cells, where the RAF pathway is constitutively activated.

With regard to polymorphisms analyses, the results indicated a patient genotype correlated to a worse prognosis, as we previously found [16–18], in accordance with the absence of response observed in this patient’s HCC lesion.

In conclusion, our results suggest that sorafenib could be effective in *BRAF*-mutated tumors. Considering that sorafenib is able to induce clinical response in about 30 % of HCC patients, it could be worth verifying the real frequency of *BRAF* mutations in this type of cancer, and whether a higher frequency of mutation is related to sorafenib response. In addition, clinical trials that evaluate the efficacy of sorafenib in NSCLC patients carrying *BRAF* mutations would be highly beneficial.

## Abbreviations

HCC, hepatocellular carcinoma; NSCLC, non-small cell lung cancer; RCC, renal cell carcinoma
